# Minimizing the Coffee Ring Effect: Improving MALDI Dried-Drop Analysis by Sequential Spotting

**DOI:** 10.1021/jasms.6c00063

**Published:** 2026-04-08

**Authors:** Joy L. Maabadi, Arbil Lopez, Joseph H. Holbrook, Amanda B. Hummon

**Affiliations:** Department of Chemistry and Biochemistry, The Ohio State University, Columbus, Ohio 43210, United States

## Abstract

In matrix-assisted laser desorption/ionization mass spectrometry (MALDI MS) experimentation, dried-drop analyses are valuable for their ease of preparation and the generation of efficient results. They hold value in their ability to assess the ionizability of an analyte or determine whether it will fragment when subjected to the MALDI laser. However, this technique can be limited by the “coffee ring effect”, producing heterogeneity in dried drops with “sweet spots” (areas of concentrated analyte) and “dead zones” (areas with little to no analyte). The coffee ring forms when the analyte concentrates on the edge of the MALDI spot, which leads to difficulty in finding consistent signals from a sample. We demonstrate that sequentially spotting dried drops at halved and quartered pipet solution levels reduces the spot diameter and crucially enhances the signal intensity. With this technique, these concentrated areas are less prevalent and there are fewer dead zones across the droplet. This study has its limitations in sample size; therefore, further investigations will be necessary. Although it increases the sample preparation time, sequential spotting makes the MS process more robust, reducing dead zones and obtaining higher signals in this proof-of-concept study.

## Graphical abstract

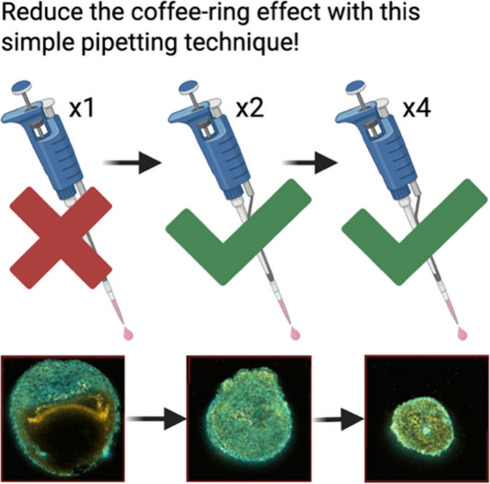

## Introduction

Matrix-assisted laser desorption/ionization mass spectrometry (MALDI MS) is a simple and powerful way to analyze biological samples, determining structure and offering characterization. Its production of singly charged ions via soft ionization allows for ease in the collection and interpretation of spectra.^1,2^ MALDI mass spectrometry imaging (MSI) expands the utility of the approach through its ability to provide information on the spatial and quantitative distribution of biomolecules.^3–5^ However, before creating any chemical heat maps of tissue samples^6–8^ or spheroids,^9,10^ analyte detection must first be established.

The most common sample preparation method for MALDI analysis is the dried-drop approach.^11–13^ Traditionally, the sample and matrix^14–16^ solutions are mixed, and a drop of resulting combined solution is pipetted onto a surface to dry for subsequent mass spectrometric analysis. This method is praised for its time-conserving nature and straightforward procedure, making it quick and easy to determine the presence of an analyte. One limitation of this method is the “coffee ring effect”,^17–23^ creating an uneven distribution of analyte and heterogeneous matrix crystallization which can lead to difficulty in producing quantitative results. The analyte within the dried drop mimics a ring left behind by a cup of coffee on a table, concentrating on the outermost part of the drop.^24^ Sequential spotting, where the total volume of liquid is applied in multiple smaller volumes with drying occurring between applications, is an alternative to pipetting the entire volume in a single droplet. In this work, sequential spotting was used to optimize the dried-drop method with linoleic acid, an essential fatty acid, as the analyte and 9-amino­acridine (9-AA) as the matrix to homogenize the sample spot and reduce the coffee ring effect. MALDI-MS imaging and subsequent statistical analyses were used to compare the effectiveness of sequential spotting in dried drops versus traditional dried-drop analysis.

## Methods

In this experiment, separate solutions of 5 mg/mL 9-AA in acetonitrile/water (75:25, v/v) and 200 μg/mL linoleic acid in methanol were combined in a 1:1 volume. 9-AA is vibrant yellow in color, which made the dried-drops stand out against the clear slides. Additionally, this color made it easier to define the clock-face positions. Three drops were analyzed, all with a total volume of 2 μL. The first was spotted with 2 μL at once, the second with two 1 μL drops, and the third with four 0.5 μL drops, with the last two conditions having allowed 5 min to dry after each drop. Seven replicates of each condition were completed, with six being technical replicates and the seventh on a different day. An additional drop was analyzed as a control (CTRL), containing 9-AA matrix alone. The control was spotted with 2 μL at once, two 1 μL, and four 0.5 μL, with 5 min of drying after each application. The drops were spotted on three separate indium–tin oxide (ITO)-coated slides and analyzed on a Bruker timsTOFfleX in negative ion mode at an *m*/*z* range of 200–1200. ITO slides were chosen for this experiment as they are most commonly used in MALDI imaging workflows. Also, on the ITO slides, it was possible to spread the samples out with the pipet (keeping the dried drops separate). Circles were drawn with a bright marker on the non-ITO-coated side of the slides to provide visual guidance during dried-drop application. Spectra were collected from the same positions in each spot, using theoretical clock positions to keep track of location. Samples were first analyzed at 10 o’clock, 2 o’clock, 4 o’clock, 8 o’clock, and center. Following the collection of the spectra at the predefined clock-face positions, the dried drops were then imaged completely via MALDI-MSI (Figure 1A and 1B). The instrument laser parameters are as follows: 1 burst of 500 shots at a frequency of 10,000 Hz, laser power 65%, and beam scan field size 20 μm × 20 μm, where the laser followed an ordered “snake”-shaped pattern.

**Figure 1:**
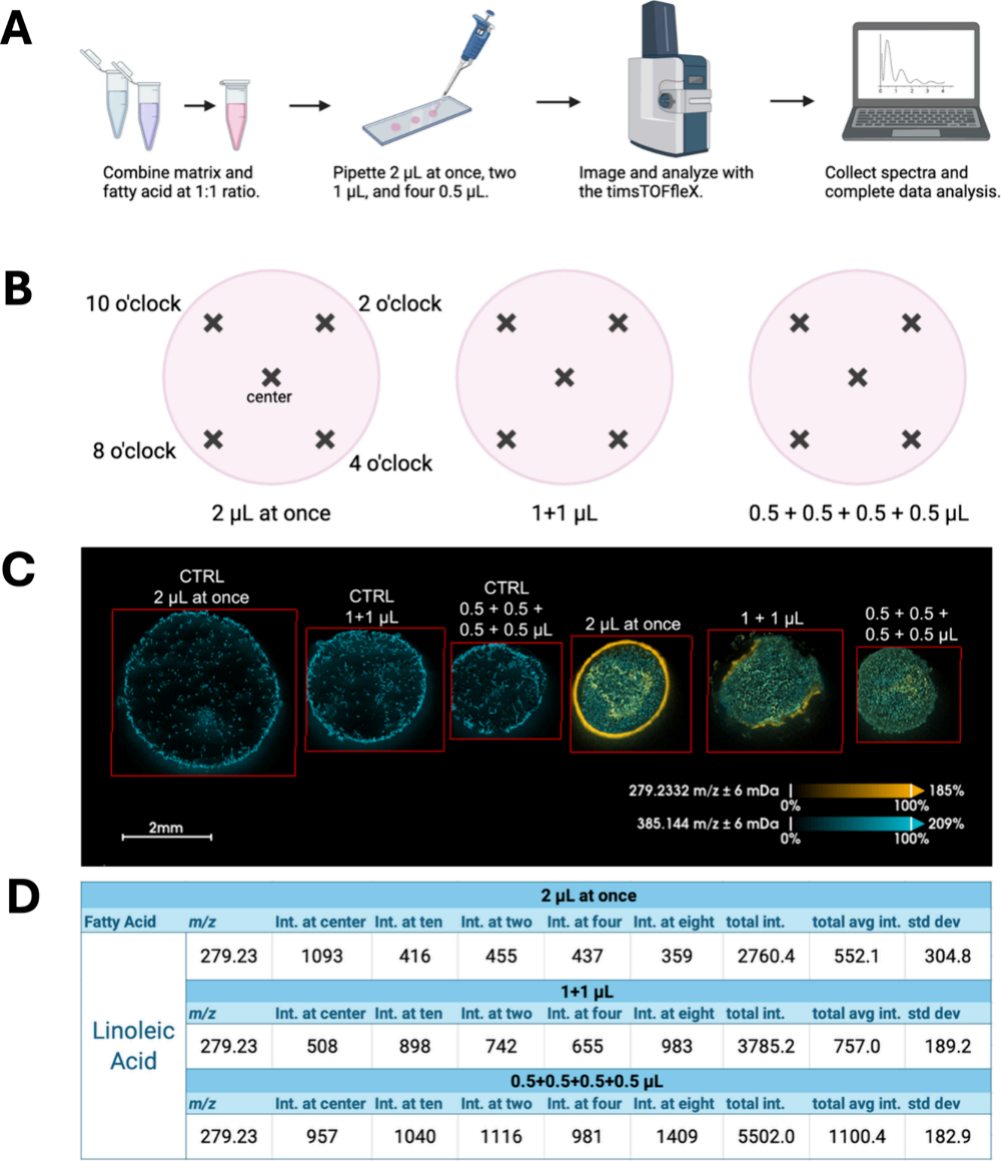
Sequential dried-drop experiment. (A) Workflow of experiment including sample preparation of dried-drop solution with 9-AA and linoleic acid, analyzed via MALDI-MSI. (B) A theoretical clock face was superimposed on dried drops, and data analysis was done according to this clock. Sampling was performed in each drop at 10 o’clock, 2 o’clock, 4 o’clock, 8 o’clock, and center, *n* = 5. (C) Images captured by MALDI-MSI. Linoleic acid (279.2332 *m*/*z*) is represented in orange and a 9-AA peak (385.144 *m*/*z*) in blue. SMART reporting standards:^25^ (S) 16 μm × 16 μm; 20 μm (beam scan setting); CTRL 2 μL: 39263 scans, CTRL 1 μL: 21661 scans, CTRL 0.5 μL: 13618 scans, 2 μL: 17944 scans, 1 μL: 21205 scans, 0.5 μL: 12652 scans, (M) MS1–0.88 ppm, (A) targeted, (R) 4000 (calculated at the linoleic acid peak), and (T) ∼30 min/ROI. (D) Values obtained from the collected spectra, where the intensities were summed and averaged and the standard deviation was found.

## Results and Discussion

In Figure 1C, linoleic acid is indicated in orange, and the matrix peak is visualized in blue. The MALDI images obtained show the pronounced coffee ring for the samples under the 2 μL-at-once condition (Figure 1C). In contrast, the dried drop produced with four applications of 0.5 μL of solution is more homogeneous and indicates no coffee ring, but rather an even distribution of analyte and matrix. The data indicates that a progression of technique (from pipetting with 2 μL to sequentially spotting the dried drops) leads to the coffee ring effect being visibly reduced, achieving a more homogeneous spread of linoleic acid. By reducing the formation of “sweet spots” (regions within the sample that are higher in concentration of analyte) in decreasing volume and increasing frequency, there are several advantages to these analyses.

The first advantage is a reduction in spot diameter as we progress from 2 to 0.5 μL, as seen in the technical replicates. Reduced spot size is beneficial because it reduces the chances of mixing between adjacent samples. On MALDI plates and ITO slides, keeping the solutions separate can prove to be a challenge, depending on the solvent of choice. Additionally, the signal intensity increases in the 1 + 1 μL and 0.5 + 0.5 + 0.5 + 0.5 μL samples compared to the single 2 μL application, demonstrated in the values from Supplemental Table 1, obtained through the clock analysis. Although the conditions had the same total amount of analyte on each drop, the application approach resulted in significant differences. Finally, Supplemental Figure 1 shows a reduction of “dead zones” (regions within the sample that are noticeably lower in concentration of analyte) across the drops, leading to ease of finding sweet spots.

While sequential spotting enhances the homogeneity and resulting signal from dried drops, it should be noted that it increases the sample preparation time since the droplets need to dry in between applications. If users are applying the standard 2 μL total spot size, pipetting **two 1 μL** or four 0.5 μL portions at a time will double or quadruple the sample preparation time. Thus, users will need to evaluate the parameters of their experiment and determine whether analysis time or signal/noise ratio is the most important figure of merit in their experiment. Finally, there are methodological limitations in this preliminary study. The use of a single matrix and analyte is a small sample size, and the results do not encompass the entirety of the dried-drop experiments. Looking forward, the universality of this trend and its conclusions will need to be investigated with additional matrices and analyte combinations.

## Conclusion

Dried-drop experiments are a simple and efficient way to begin MALDI imaging experiments but bring some difficulty when the analyte of interest forms either a coffee ring or dead zones within the sample. A practical solution to this problem is to increase the number of applications while using lower volumes to maintain a consistent concentration, thereby reducing the coffee ring effect and streamlining analysis. For matrix 9-AA and the analyte linoleic acid, a practical solution is to increase the number of applications while using lower volumes to maintain a consistent concentration, thereby reducing the coffee ring effect and streamlining analysis. This initial proof-of-concept study demonstrates that sequentially spotting dried drops by reducing the pipet volume to either 1 μL or 0.5 μL works to increase the homogeneity of the dried drop and increases the signal intensity in the drop for this matrix/analyte combination. We are increasing the reproducibility of dried drops by providing greater shot-to-shot consistency while removing the necessity of searching for these sweet spots. The resulting spectra collected can therefore be accepted as more accurate and reliable and received with less ambiguity. Assurance is gained by seeing that the instrument of choice has the sensitivity to detect analytes that typically prove to be difficult to track. For the sake of minimizing the nuisance of searching for sweet spots or avoiding dead zones, we have found that it is worth pipetting twice or four times over to improve the analysis, allowing the experimenter to focus on other aspects of their work.

## Supplementary Material



## Notes

Supplemental Figure 1. MALDI MSI imaging of Ssx replicates of the sequential spotting experiment. Supplemental Table 1. Summary Table of the total average intensities found at each condition from the data in Supplemental Figure 1. Supplemental Table 2. Signal intensities collected for each of the technical replicates shown in Supplemental Figure 1 (PDF)

## Abbreviations

MALDI-MSI: matrix-assisted laser desorption/ionization mass spectrometry imaging
9-AA: 9-aminoacridine
ITO: indium–tin oxide

## References

[1] El-Aneed A., Cohen A., Banoub J. (2009). Mass Spectrometry, Review of the
Basics: Electrospray, MALDI, and Commonly Used Mass Analyzers. Appl. Spectrosc. Rev..

[2] Hillenkamp, F. ; Jaskolla, T. W. ; Karas, M. The MALDI Process and Method. In MALDI MS: A Practical Guide to Instrumentation, Methods, and Applications, 2nd ed.; Hillenkamp, F. , Peter-Katalinic, J. , Eds.; John Wiley & Sons, Ltd, 2013; pp 1–40. DOI: 10.1002/9783527335961.ch1.

[3] Lopez A., Holbrook J. H., Kemper G. E., Lukowski J. K., Andrews W. T., Hummon A. B. (2024). Tracking Drugs and
Lipids: Quantitative Mass Spectrometry
Imaging of Liposomal Doxorubicin Delivery and Bilayer Fate in Three-Dimensional
Tumor Models. Anal. Chem..

[4] MALDI-MSI and Spatial Analysis. https://www.labxchange.org/library/items/lb:LabXchange:174a3c88:html:1 (accessed 2026-02-12).

[5] Tuck M., Grélard F., Blanc L., Desbenoit N. (2022). MALDI-MSI
Towards Multimodal Imaging: Challenges and Perspectives. Front. Chem..

[6] Grgic A., Krestensen K. K., Heeren R. M. A. (2023). Optimized Protocol for MALDI MSI
of N-Glycans Using an on-Tissue Digestion in Fresh Frozen Tissue Sections. Sci. Rep..

[7] Høiem T. S., Andersen M. K., Martin-Lorenzo M., Longuespée R., Claes B. S. R., Nordborg A., Dewez F., Balluff B., Giampà M., Sharma A., Hagen L., Heeren R. M. A., Bathen T. F., Giskeødegård G. F., Krossa S., Tessem M.-B. (2022). An Optimized MALDI MSI Protocol for
Spatial Detection
of Tryptic Peptides in Fresh Frozen Prostate Tissue. Proteomics.

[8] Mascini N. E., Teunissen J., Noorlag R., Willems S. M., Heeren R. M. A. (2018). Tumor
Classification with MALDI-MSI Data of Tissue Microarrays: A Case Study. Methods.

[9] Fries B. D., Hummon A. B. (2025). Mass Spectrometry–Based Applications
of Spheroids
in Cancer Biology. Annual Review of Analytical
Chemistry.

[10] Wang Y., Hummon A. B. (2023). Quantification of Irinotecan in Single Spheroids Using
Internal Standards by MALDI Mass Spectrometry Imaging. Anal. Chem..

[11] Hu H., Larson R. G. (2002). Evaporation of a
Sessile Droplet on a Substrate. J. Phys. Chem.
B.

[12] Ou Y.-M., Tsao C.-W., Lai Y.-H., Lee H., Chang H.-T., Wang Y.-S. (2016). Preparation of Homogeneous MALDI Samples for Quantitative
Applications. J. Vis. Exp..

[13] van
Kampen J. J. A., Burgers P. C., de Groot R., Gruters R. A., Luider T. M. (2011). Biomedical Application of MALDI Mass Spectrometry for
Small-Molecule Analysis. Mass Spectrom. Rev..

[14] Cohen S. L., Chait B. T. (1996). Influence of Matrix
Solution Conditions on the MALDI-MS
Analysis of Peptides and Proteins. Anal. Chem..

[15] Mielczarek P., Suder P., Kotsan I., Bodzon-Kulakowska A. (2023). The Influence
of Matrix Concentration and Solvent Composition on the Results of
MALDI MSI, with the Aid of Wet-Interface Matrix Deposition. J. Mass Spectrom..

[16] Gusev A. I., Wilkinson W. R., Proctor A., Hercules D. M. (1995). Improvement of Signal
Reproducibility and Matrix/Comatrix Effects in MALDI Analysis. Anal. Chem..

[17] Zeng Z., Wang Y., Shi S., Wang L., Guo X., Lu N. (2012). On-Plate Selective
Enrichment and Self-Desalting of Peptides/Proteins
for Direct MALDI MS Analysis. Anal. Chem..

[18] Blossey R., Bosio A. (2002). Contact Line Deposits
on cDNA Microarrays: A “Twin-Spot Effect”. Langmuir.

[19] Deegan R. D., Bakajin O., Dupont T. F., Huber G., Nagel S. R., Witten T. A. (1997). Capillary Flow as
the Cause of Ring Stains from Dried
Liquid Drops. Nature.

[20] Eral B., Van den Ende D., Mugele F. (2012). Say Goodbye to Coffee Stains. Phys. World.

[21] Hu H., Larson R. G. (2006). Marangoni
Effect Reverses Coffee-Ring Depositions. J.
Phys. Chem. B.

[22] Hu J.-B., Chen Y.-C., Urban P. L. (2013). Coffee-Ring
Effects in Laser Desorption/Ionization
Mass Spectrometry. Anal. Chim. Acta.

[23] Kudina O., Eral B., Mugele F. (2016). E-MALDI: An
Electrowetting-Enhanced
Drop Drying Method for MALDI Mass Spectrometry. Anal. Chem..

[24] Wu M., Man X., Doi M. (2018). Multi-Ring Deposition Pattern of
Drying Droplets. Langmuir.

[25] Xi Y., Sohn A. L., Joignant A. N., Cologna S. M., Prentice B. M., Muddiman D. C. (2023). SMART: A Data Reporting
Standard for Mass Spectrometry
Imaging. J. Mass Spectrom,.

