# Supplementary material for: Minimizing the Coffee Ring Effect: Improving MALDI Dried-Drop Analysis by Sequential Spotting
Source: J Am Soc Mass Spectrom. 2026 Apr 8;37(5):1064–7. doi: 10.1021/jasms.6c00063 (PMC13154212; doi:10.1021/jasms.6c00063)
Supplement: Supplementary file 1 [file js6c00063_si_001.pdf]

Supporting Information

Minimizing the Coffee Ring Effect: Improving MALDI Dried Drop Analysis by Sequential Spotting

*Joy L. Maabadi, Arbil Lopez, Joseph H. Holbrook, Amanda B. Hummon**

Author Address: Department of Chemistry and Biochemistry, The Ohio State University, Columbus, Ohio 43210, United States

Corresponding Author: Amanda B. Hummon (hummon.1@osu.edu) - Department of Chemistry and Biochemistry, The Ohio State University, Columbus, Ohio 43210, United States

Supporting Information

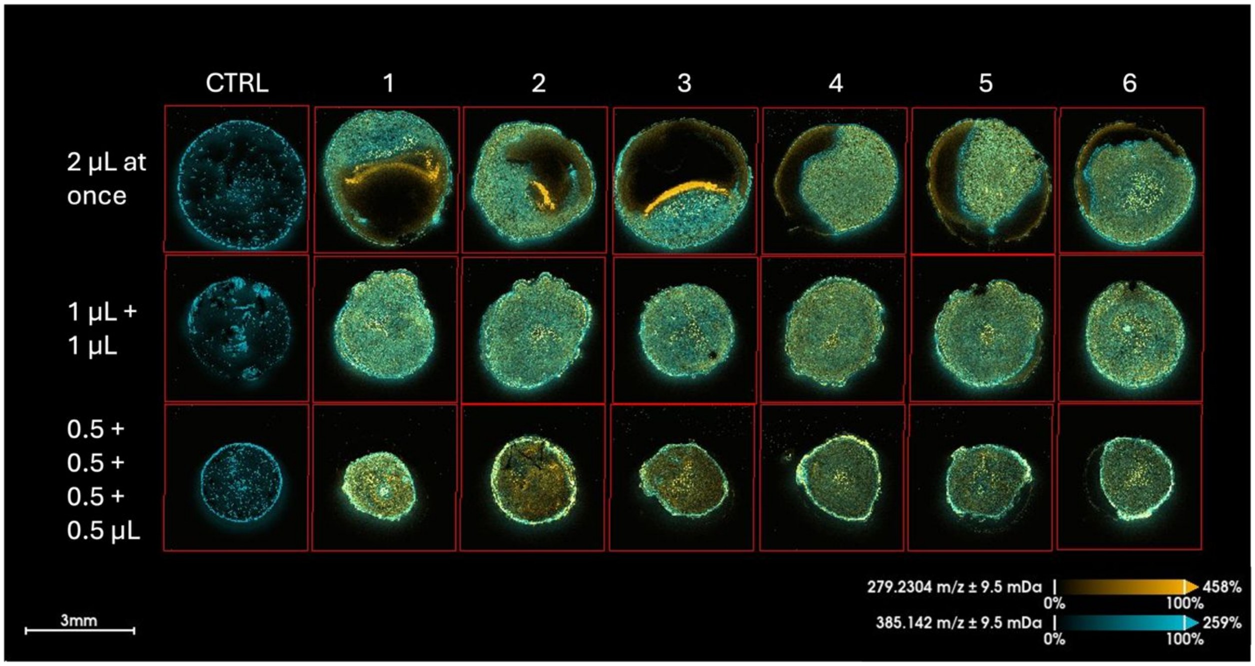

Supplemental Figure 1. Six replicates of the sequential spotting experiment, each number representing a technical replicate. Images captured by MALDI-MSI. Linoleic acid (279.2304 *m/z*) is represented in orange and a 9-AA peak (385.144 *m/z*) in blue. SMART reporting standards: (S) 16 µm × 16 µm; 20 µm (beam scan setting); 40,621 scans/square, (M) MS1–10 ppm, (A) targeted, (R) 3846 (calculated at the linoleic acid peak), and (T) ~90 min/ROI.

Summary Table, Total Average Intensity, n=6				
Fatty Acid	*m/z*	2 μL at once	1+1 μL	0.5+0.5+0.5+0.5 μL
Linoleic Acid	279.23	2630.5	3521.2	4579.1

Supplemental Table 1. Summary Table of the total average intensities found at each condition from the data in Supplemental Figure 1.

				Replicate 1: 2 µL at once					
Fatty Acid	*m/z*	Int. at center	Int. at ten	Int. at two	Int. at four	Int. at eight	total int.	total avg int.	std dev
Linoleic Acid	279.23	9	484	72	21	197	783.4	156.7	197.4
				1+1 µL					
	m/z	Int. at center	Int. at ten	Int. at two	Int. at four	Int. at eight	total int.	total avg int.	std dev
	279.23	1182	655	776	352	483	3447	689.4	319.3
				0.5+0.5+0.5+0.5 µL					
	m/z	Int. at center	Int. at ten	Int. at two	Int. at four	Int. at eight	total int.	total avg int.	std dev
	279.23	2545	673	727	808	705	5457.4	1091.5	814.0
				Replicate 2: 2 µL at once					
Fatty Acid	*m/z*	Int. at center	Int. at ten	Int. at two	Int. at four	Int. at eight	total int.	total avg int.	std dev
Linoleic Acid	279.23	590	462	51	804	556	2462.4	492.5	276.9
				1+1 µL					
	m/z	Int. at center	Int. at ten	Int. at two	Int. at four	Int. at eight	total int.	total avg int.	std dev
	279.23	1271	593	358	547	513	3282	656.4	354.7
				0.5+0.5+0.5+0.5 µL					
	m/z	Int. at center	Int. at ten	Int. at two	Int. at four	Int. at eight	total int.	total avg int.	std dev
	279.23	886	851	1120	687	852	4395.0	879.0	155.2
				Replicate 3: 2 µL at once					
Fatty Acid	*m/z*	Int. at center	Int. at ten	Int. at two	Int. at four	Int. at eight	total int.	total avg int.	std dev
Linoleic Acid	279.23	981	3	0	280	713	1977.2	395.4	438.1
				1+1 µL					
	m/z	Int. at center	Int. at ten	Int. at two	Int. at four	Int. at eight	total int.	total avg int.	std dev
	279.23	217	558	555	229	426	1985	396.9	167.5
				0.5+0.5+0.5+0.5 µL					
	m/z	Int. at center	Int. at ten	Int. at two	Int. at four	Int. at eight	total int.	total avg int.	std dev
	279.23	970	592	536	518	938	3554.2	710.8	223.6
				Replicate 4: 2 µL at once					
Fatty Acid	*m/z*	Int. at center	Int. at ten	Int. at two	Int. at four	Int. at eight	total int.	total avg int.	std dev
Linoleic Acid	279.23	519	207	860	619	564	2769.2	553.8	234.3
				1+1 µL					
	m/z	Int. at center	Int. at ten	Int. at two	Int. at four	Int. at eight	total int.	total avg int.	std dev
	279.23	478	672	528	568	505	2752	550.4	75.9
				0.5+0.5+0.5+0.5 µL					
	m/z	Int. at center	Int. at ten	Int. at two	Int. at four	Int. at eight	total int.	total avg int.	std dev
	279.23	1622	702	422	523	493	3761.4	752.3	496.8
				Replicate 5: 2 µL at once					
Fatty Acid	*m/z*	Int. at center	Int. at ten	Int. at two	Int. at four	Int. at eight	total int.	total avg int.	std dev
Linoleic Acid	279.23	771	91	657	771	653	2942.2	588.4	284.2
				1+1 µL					
	m/z	Int. at center	Int. at ten	Int. at two	Int. at four	Int. at eight	total int.	total avg int.	std dev
	279.23	442	430	482	406	518	2277	455.4	44.4
				0.5+0.5+0.5+0.5 µL					
	m/z	Int. at center	Int. at ten	Int. at two	Int. at four	Int. at eight	total int.	total avg int.	std dev
	279.23	556	410	480	587	236	2269.8	454.0	140.1
				Replicate 6: 2 µL at once					
Fatty Acid	*m/z*	Int. at center	Int. at ten	Int. at two	Int. at four	Int. at eight	total int.	total avg int.	std dev
Linoleic Acid	279.23	261	642	383	452	481	2218.2	443.6	139.5
				1+1 µL					
	m/z	Int. at center	Int. at ten	Int. at two	Int. at four	Int. at eight	total int.	total avg int.	std dev
	279.23	1724	419	508	827	386	3864	772.8	559.6
				0.5+0.5+0.5+0.5 µL					
	m/z	Int. at center	Int. at ten	Int. at two	Int. at four	Int. at eight	total int.	total avg int.	std dev
	279.23	1402	477	551	434	595	3457.6	691.5	402.1

Supplemental Table 2. Signal intensities collected for each of the technical replicates shown in Supplemental Figure 1.
